# Correction: Updated cost-effectiveness of MDMA-assisted therapy for the treatment of posttraumatic stress disorder in the United States: Findings from a phase 3 trial

**DOI:** 10.1371/journal.pone.0269623

**Published:** 2022-06-02

**Authors:** Elliot Marseille, Jennifer M. Mitchell, James G. Kahn

The Data Availability statement for this paper is incorrect. The correct statement is: The data that support the findings of this study are available from the sponsor (MAPS). However, restrictions apply to the availability of these data, which were used under license for the current study, and so are not publicly available. Data are, however, available from the authors upon reasonable request and with the permission of MAPS at http://maps.org/datause. All requests for raw and analyzed data are promptly reviewed by the sponsor delegate and trial organizer, MAPS PBC, to verify if the request is subject to any confidentiality obligations. Patient-related data not included in the paper were generated as part of clinical trials and may be subject to patient confidentiality. Any data that can be shared will be released via a data use agreement. In accessing the data from MAPS, the authors of this study did not receive any special privileges which other researchers would not have.
