# Supplementary material for: Updated cost-effectiveness of MDMA-assisted therapy for the treatment of posttraumatic stress disorder in the United States: Findings from a phase 3 trial
Source: PLoS One. 2022 Feb 25;17(2):e0263252. doi: 10.1371/journal.pone.0263252 (PMC8880875; doi:10.1371/journal.pone.0263252)
Supplement: S1 File — (PDF) [file pone.0263252.s001.pdf]

Contents

eMethods A: Classification of patients by PTSD severity category using CAPS-5 scores. 1

eMethods B: CPT codes of intervention costs 2

eMethods A: Classification of patients by PTSD severity category using CAPS-5 scores.

A validation study of the CAPS-5 scale defined the range for moderate PTSD as 23 to 34 for CAPS 5, and 40 to 59 for CAPS IV[1]. The full set of severity categories for CAPS-IV are shown in the table below[2]. Severe PTSD would therefore begin at 35 and 60 for CAPS-5 and CAPS-IV, respectively. We applied the ratio between these numbers (35/60 = 0.5833), to the CAPS-IV ranges for each category, and rounded to the nearest unit to avoid overlapping categories,

Relationship between CAPS-IV, CAPS-5 scores and PTSD severity

	CAPS-IV	CAPS-5
Asymptomatic	0-19	0-11
Mild	20-39	12-22
Moderate	40-59	23-34
Severe	60-79	35-47
Extreme	80+	48+

References

1. Weathers FW, Bovin MJ, Lee DJ, et al. The Clinician-Administered PTSD Scale for DSM-5 (CAPS-5): Development and initial psychometric evaluation in military veterans. *Psychol Assess*. 2018;30(3):383-395. doi:10.1037/pas0000486
2. Weathers FW, Keane TM, Davidson JR. Clinician-administered PTSD scale: a review of the first ten years of research. *Depress Anxiety*. 2001;13(3):132-56. doi:10.1002/da.1029

eMethods B: CPT codes of intervention costs

Current Procedural Terminology (CPT) codes were assigned to each MAT activity. These included CPT code 90837 for psychotherapy, pro-rated over the 90 minute length of the ‘pre’ and ‘post’ sessions and the 8 hours of the MDMA sessions; CPT codes 96130 and 90792 for intake and screening respectively; and, for the 10% of patients who, due to potential cardiac risk identified at screening, required nuclear stress tests and carotid ultrasound, 93015 and 93880 respectively. Two therapists participated in all sessions. The cost associated with CPT codes 90837and 90792 was assigned the average cost of eight metropolitan areas as provided by *FAIR Health Consumer* which maintains a large private and public payer national claims data base[1]. Costs associated with CPT codes 96130, 93015 and 93880, are Medicare allowable reimbursement amount as provided by the Centers for Medicare and Medicaid Services[2].

References

1. FAIR Health Consumer. Search for medical and hospital costs. https://www.fairhealthconsumer.org/
2. Centers for Medicaid and Medicare Services. Physician Fee Schedule Search,. Accessed March 15, 2020, 2020. https://www.cms.gov/apps/physician-fee-schedule/license-agreement.aspx
